# Charting New Territory: AI Applications in Dental Caries Detection from Panoramic Imaging

**DOI:** 10.3390/dj13080366

**Published:** 2025-08-12

**Authors:** Man Hung, Daniel Yevseyevich, Milan Khazana, Connor Schwartz, Martin S. Lipsky

**Affiliations:** 1 College of Dental Medicine, Roseman University of Health Sciences, South Jordan, UT 84095, USA; 2 Division of Public Health, University of Utah, Salt Lake City, UT 84108, USA; 3 School of Business, University of Utah, Salt Lake City, UT 84112, USA; 4 Primary Children’s Hospital, Salt Lake City, UT 84113, USA; 5 Library, Roseman University of Health Sciences, South Jordan, UT 84095, USA; 6 Library, Noorda College of Osteopathic Medicine, Provo, UT 84606, USA; 7 Institute on Aging, Portland State University, Portland, OR 97207, USA

**Keywords:** panoramic radiograph, AI programs, healthcare, dentistry

## Abstract

**Introduction:** Dental caries remains a public health concern, and early detection prevents its progression and complications. Panoramic radiographs are essential diagnostic tools, yet the interpretation of panoramic X-rays varies among practitioners. Artificial intelligence (AI) presents a promising approach to enhance diagnostic accuracy in detecting dental caries. This scoping review examines the current literature on the use of AI programs to analyze panoramic radiographs for the diagnosis of dental caries. **Methods:** This scoping review searched PubMed, Scopus, Web of Science, and Dentistry and Oral Sciences Source, adhering to PRISMA guidelines. The review included peer-reviewed, original research published in English that investigated the use of AI to diagnose dental caries. Data were extracted on the AI model characteristics, advantages, disadvantages, and diagnostic performance. **Results:** Seven studies met the inclusion criteria. The Deep Learning Model achieved the highest performance (specificity 0.9487, accuracy 0.9789, F1 score 0.9245), followed by Diagnocat and Tooth Type Enhanced Transformer. Models such as CranioCatch and CariSeg showed moderate performance, while the Dental Caries Detection Network demonstrated the lowest. Benefits included improved diagnostic support and workflow efficiency, while limitations involved dataset biases, interpretability challenges, and computational demands. **Conclusions:** Applying AI technologies to panoramic X-rays demonstrates the potential for enhancing caries diagnosis, with some models achieving near-expert performance. However, future research must address the generalizability, transparency, and integration of AI models into clinical practice. Future research should focus on diverse training datasets, explainable AI development, clinical validation, and incorporating AI training into dental education and training.

## 1. Introduction

Dental caries can significantly impair an individual’s quality of life by causing pain, irritation, sensitivity, tooth loss, and functional impairment [1,2]. Despite their preventability, carious lesions are the most common health condition in the world, and if left untreated, may progress to disorders that require more extensive and costly interventions compared to the treatment of the original lesion [3]. Consequently, secondary prevention [4,5], or the early detection of caries, enables timely intervention to halt disease progression and maintain oral health without residual pathology [5].

Using a traditional visual-tactile examination may miss hidden or less accessible carious lesions, making radiographic imaging essential for comprehensive detection [4]. Dentists commonly use panoramic, periapical, and bitewing radiographs to augment their clinical assessment for diagnosing caries [4]. Panoramic radiography visualizes the maxillomandibular area on a single film and, since its introduction, has gained popularity and value as a diagnostic tool in general dentistry. Panoramic radiography examines the whole dentition, alveolar bone, temporomandibular joints, and surrounding tissues and since it identifies pathologies not readily apparent during a clinical examination such as subgingival calculus, furcation involvement, and root surface caries, it is used for patient screening at several institutions and private clinics [6,7].

While panoramic radiography offers a comprehensive view of the maxillofacial structures, it has notable limitations for detecting dental caries. The major drawback of taking panoramic radiographs is that it requires the digital post-processing of panoramic images to provide the most accurate presentation of caries [8,9]. As a result, panoramic radiographs have traditionally been considered less reliable for caries detection, especially in the posterior interproximal regions. However, technological advancements have introduced panoramic systems capable of producing bitewing-style images, which have improved diagnostic capability in detecting interproximal caries [10]. Moreover, the visualization of moderate to advanced lesions on panoramic radiographs, particularly in good-quality images, is an area of growing interest to supplement intraoral imaging when it is limited or not feasible [11,12]. This potential was enhanced by the integration of artificial intelligence (AI), which can aid in standardizing lesion detection and potentially compensate for some of PR’s limitations. Therefore, while PR may not be the gold standard for caries diagnosis, its evolving role in opportunistic and AI-assisted caries detection warrants systematic evaluation.

In recent years, AI—particularly machine learning and deep learning—has emerged as a powerful tool that facilitates the diagnosis of conditions that might be challenging even for experienced dentists [13]. By automating the detection and classification of dental caries, AI has the potential to reduce diagnostic errors, increase consistency among practitioners, and improve the overall efficiency of dental care delivery [14]. AI systems can process large amounts of radiographic data, learning to identify patterns associated with dental caries and other oral conditions with high precision [14]. Clinical studies demonstrate that AI models can achieve diagnostic performance and serve as a dependable second option to experienced clinicians [15]. Additionally, AI performance heavily depends on the quality and diversity of the training dataset, and a limited training dataset may also not account for all anatomical or pathological variations found within a patient population [15].

The rapid advancements in AI technology and its growing application in dental diagnostics make it important to conduct a comprehensive evaluation of using AI in conjunction with panoramic radiographs for caries diagnosis. Although other reviews examined the use of AI technology and dental imaging in diagnosing caries [15,16,17], this is the first review to explore the application of AI in diagnosing caries on panoramic X-rays. A scoping review is particularly well-suited for this purpose, as it maps the current literature, identifies research gaps, and provides direction for future investigations.

Therefore, the objective of this scoping review was to systematically investigate the current landscape of AI applications designed to detect dental caries using panoramic radiographs. By critically analyzing the reported strengths and limitations of various AI learning models, this review offers a comprehensive overview of their diagnostic capabilities and practical implications. In doing so, it aims to inform clinicians, researchers, and policymakers on how best to integrate AI into dental workflows in a manner that enhances diagnostic accuracy without compromising clinical judgment or patient care. The review placed a special emphasis on evaluating the diagnostic performance of different AI systems, highlighting their potential to significantly improve the early and accurate detection of caries in clinical settings. This review not only maps current advancements but also identifies critical gaps in the literature that warrant future investigation.

## 2. Methods

### 2.1. Criteria Selection

This scoping review followed the PRISMA guidelines for scoping reviews [18]. The objective was to comprehensively map the existing literature on the advantages and disadvantages of using various AI programs to analyze panoramic radiographs for diagnosing dental caries. This section outlines the procedures used for study identification, including the search strategy, eligibility criteria, study selection process, data extraction, and synthesis.

The reviewers conducted a comprehensive literature search across four electronic databases—PubMed, Scopus, Web of Science, and Dentistry and Oral Sciences Source—to identify relevant studies published up to September 2024. The search strategy, developed in collaboration with a medical librarian, combined controlled vocabulary terms and free-text keywords related to artificial intelligence, panoramic radiography, dental caries, diagnosis, advantages, and disadvantages. The search used Boolean operators to ensure comprehensive retrieval of relevant literature and an English-language filter across all databases. Keywords and MeSH terms included “artificial intelligence”, “machine learning”, “deep learning”, “panoramic radiography”, “dental caries”, and “diagnosis”. The search yielded a total of 64 records: 17 from PubMed, 33 from Scopus, 8 from Web of Science, and six from Dentistry & Oral Sciences Source. Table 1 summarizes the query used for screening.

### 2.2. Inclusion and Exclusion Criteria

Table 2 summarizes the eligibility criteria used for screening. The review included peer-reviewed, English language, original human research studies that specifically investigated the application of AI to diagnose dental caries using panoramic radiographs, available as full-text articles. The review excluded studies that did not examine panoramic AI and dental caries diagnosis or were literature reviews (narrative, scoping, or systematic), conference abstracts, editorials, opinion pieces, or were unavailable as full-text or in English. Additionally, the review excluded basic research and animal studies. These stringent criteria minimize bias, enhance comparability across studies, and ensure the synthesized evidence has direct clinical applicability within AI-driven dental diagnostics.

### 2.3. Abstract Screening and Data Extraction

The reviewers performed study selection in two stages: (1) title and abstract screening, followed by (2) full-text review. In the first stage, two independent reviewers (D.Y. and M.H.) evaluated and screened the titles and abstracts of all retrieved articles against the eligibility criteria, excluding research papers that did not meet the inclusion criteria. The reviewers resolved discrepancies through discussion. In the second stage, the full texts of potentially eligible studies were independently assessed by D.Y. and M. K. who used a standardized data extraction form that included study characteristics, AI program details, diagnostic outcomes, conclusions regarding the use of AI with panoramic radiographs for diagnosing dental caries and documented reasons for excluding a study. A third reviewer (M.H.) audited the selection process to ensure accuracy and review quality.

### 2.4. Data Synthesis

Data synthesis involved a descriptive-analytical approach to map the existing evidence on the advantages and disadvantages of applying AI to panoramic radiographs for dental caries diagnosis. Where applicable, quantitative data, such as diagnostic accuracy and precision metrics, were summarized using descriptive statistics, as well as accuracy, precision, and F1 scores.

## 3. Results

**Figure 1:**
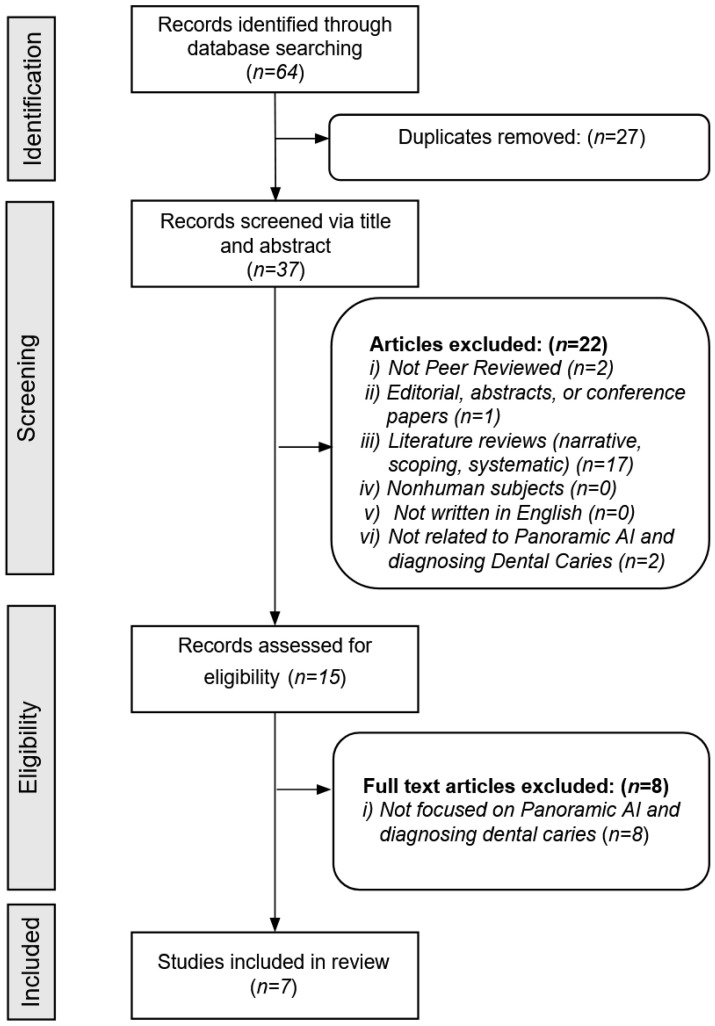
Search strategy.

Figure 1 provides a detailed overview of the article screening process. The database searches initially identified a total of 64 articles. After removing 27 duplicates, 37 unique articles remained. Based on the screening inclusion and exclusion criteria, the reviewers excluded 30 additional articles, leaving seven articles for full analysis of AI modeling data.

Table 3 summarizes the studies and their findings. The reviewed studies span multiple countries and diverse patient populations. All were conducted in university settings, with three in Turkey, two in Europe, one in China, and one multinational. The number of images evaluated ranged from 30 to 6028. The studies focused primarily on adults, except for one pediatric study. Three studies did not specify age.

Table 3 also presents a detailed comparison of the AI programs used. CranioCatch demonstrated high sensitivity in detecting crowns, implants, and impacted teeth, highlighting its efficiency and potential for educational and remote diagnostic use; however, it showed poor performance in detecting dental calculus and caries [20]. The Deep Learning-based Segmentation of Caries model improved lesion detection and enabled early intervention, but required substantial computational resources and faced generalization challenges across different datasets [19]. CariSeg enhanced diagnostic accuracy, processed large datasets rapidly, and reduced human error, although its accuracy was influenced by image quality and patient positioning [22]. Diagnocat improved clinicians’ diagnostic confidence and adapted to various imaging types, but required significant computational power and raised concerns regarding transparency and ethical accountability [12]. Diagnocat alone was unacceptable in detecting caries [23]. The Deep Learning Model (DLM) excelled in diagnosing complex dental cases and integrated well with clinical software systems, but required large and diverse training datasets and experienced delays in real-time diagnostics [23]. The Tooth Type Enhanced Transformer (TTET) prioritized model interpretability and achieved high lesion detection accuracy but faced difficulties with atypical presentations and encountered technical challenges with clinical integration [24]. Although the AI systems showed considerable promise in improving diagnostic workflows, their limitations underscore the need for further validation and clinical adaptation.

Among the AI programs evaluated (Table 4), the DLM showed the highest overall performance, with a specificity of 0.95, accuracy of 0.98, and F1 score of 0.92 [19]. Diagnocat, assessed in two separate studies, also demonstrated high specificity (0.86 and 0.98; Mean = 0.92; Standard Deviation = 0.08) and accuracy (0.88 and 0.85; Mean = 0.87; Standard Deviation = 0.02), though F1 scores varied between 0.86 and 0.59 Mean = 0.73; Standard Deviation = 0.19), respectively [12,23]. Similarly, the TTET reported strong performance, with specificity of 0.83, accuracy of 0.88, and an F1 score of 0.87 from 210 radiographs [24]. In comparison, CariSeg achieved moderate results, with a specificity of 0.51, accuracy of 0.81, and an F1 score of 0.65 based on 150 radiographs [22]. DCDNet performed lower with specificity at 0.47, accuracy at 0.56, and an F1 score of 0.61 [21]. The lowest performance was observed with CranioCatch which yielded a specificity of 0.30, accuracy of 0.51, and an F1 score of 0.38 [20].

## 4. Discussion

Although bitewing and periapical dental X-rays remain the standard for detecting caries, panoramic radiographs still play an important role in diagnosing and managing caries. They identify larger cavities, especially those between teeth or under existing fillings that might be missed with other types of X-rays, and can help with planning treatment [6,7]. The findings of this scoping review illustrate the significant advancements and challenges in applying AI to assess panoramic radiographs. These AI models align with practitioners who utilize them to enhance diagnostic workflows, improve detection rates, and assist clinicians in decision-making processes [25].

Among the evaluated AI models, DLM, Diagnocat, and TTET demonstrated superior diagnostic capabilities. DLM achieved the highest average overall performance, with specificity, accuracy, and F1 scores all exceeding 0.90, indicating its robustness in correctly identifying both carious and non-carious lesions. However, not all AI programs achieved comparable levels of performance. CranioCatch and CariSeg displayed moderate metrics, and DCDNet lagged significantly behind, highlighting issues related to dataset bias, generalizability, and technical limitations. These disparities underscore the important link between diverse and comprehensive training datasets to generalizability and performance.

While AI holds promise in increasing diagnostic consistency, an overreliance on automated systems may promote an overreliance on AI-related ethical considerations also extending to data privacy and informed consent. The use of patient radiographic data to train, validate, and improve AI models must adhere to strict data governance standards. While de-identified data may mitigate privacy risks, maintaining transparency about data use and identifying any potential commercial interests involved in AI development remain essential to maintaining trust [26]. The European Union’s AI Act emphasizes the need for trustworthy AI systems, highlighting the importance of data protection and ethical considerations in AI deployment [27].

### 4.1. Clinical Implications

AI caries detection models can serve as a valuable tool to practitioners by providing feedback and a second opinion as they interpret radiographs, and to patients who might benefit from visualizing caries on a radiograph that would normally require training to interpret. Interdisciplinary collaboration between dental practitioners, data scientists, ethicists, and policymakers is crucial to ensure that AI systems are not only technically effective but also socially responsible and patient-centered [27]. However, designers should consider AI deployment strategies. Systems should include feedback loops that enable clinicians to verify, contest, or refine AI-suggested diagnoses.

### 4.2. Strengths and Limitations

This review has several strengths, including the use of a systematic and comprehensive search strategy, clearly defined inclusion and exclusion criteria, and a focus on peer-reviewed, full-text original research involving human subjects. The use of consistent performance metrics (specificity, accuracy, and F1 scores) across studies enabled meaningful comparisons between AI programs.

However, several limitations should be acknowledged. The inclusion criteria limited the review to articles published in English, possibly excluding relevant studies in other languages. There was also considerable heterogeneity among studies in terms of the specific AI architecture employed, dataset characteristics, and performance evaluation methods. As a result, while this review could identify general patterns, a direct meta-analytical comparison was not feasible. Not all studies included the age ranges for the population x-rayed and might compromise comparisons. Furthermore, the rapid evolution of AI technologies means that studies up to 2024 may not capture the latest innovations.

### 4.3. Future Directions

Future research should prioritize the development of larger, multi-institutional, and diverse datasets to train AI models. Current models often suffer from limited generalizability due to the homogeneity of their training data, which can decrease diagnostic performance across varied clinical populations [28].

Although augmented intelligence and AI have been available for decades, dentistry has only recently integrated these modalities into clinical practice and dental education and training. AI has the potential to improve student learning in preclinical and clinical training, according to early examples of its integration into dental schools [29]. AI can support dental practitioners’ clinical decision-making, which might improve the accuracy and consistency of radiographic interpretation [30]. By embedding AI literacy and training into dental education programs, the profession can ensure that it implements emerging technologies correctly to enhance clinical practice while maintaining a high standard of patient-centered care.

### 4.4. Conclusions

In conclusion, AI-supported diagnostic programs offer exciting opportunities to improve dental caries detection using panoramic radiographs. Models such as DLM and Diagnocat demonstrate that, with appropriate design and training, AI can match or even surpass human diagnostic capabilities. Nevertheless, significant challenges remain related to generalizability, interpretability, ethical considerations, and clinical integration. Maximizing the advantages of AI while mitigating its risks will rely on continued research, interdisciplinary collaboration, and implementation strategies. AI is poised to become an indispensable part of dental diagnostics—provided that its deployment remains grounded in a commitment to enhancing, not replacing, clinical expertise and patient-centered care.

## Figures and Tables

**Table 1:** Query used for screening.

Database Date	Search Strategy Filters	Results
PubMed 20 June 2024	(“Artificial Intelligence”[Mesh] OR “Artificial intelligence” OR “AI” OR “Computer Reasoning” OR “Machine Intelligence” OR “Deep Learning”) AND (“Dental Caries”[Mesh] OR “Dental Caries/diagnostic imaging”[Mesh] OR “Dental Caries” OR “Dental Decay” OR “Dental Cavities” OR “Carious Lesion” OR “Carious Lesions” OR “Dental Cavity”) AND (“Radiography, Panoramic”[Mesh] OR “Panoramic radiograph” OR “Panoramic Radiography” OR “Panoramic Radiographies” OR “Orthopantomography” OR “Orthopantomographies” OR “Pantomography” OR “Pantomographies”) Filter: English	17
Scopus 20 June 2024	TITLE-ABS-KEY ((“Artificial intelligence” OR “AI” OR “Computer Reasoning” OR “Machine Intelligence” OR “Deep Learning”) AND (“Dental Caries” OR “Dental Decay” OR “Dental Cavities” OR “Carious Lesion” OR “Carious Lesions” OR “Dental Cavity”) AND (“Panoramic radiograph” OR “Panoramic Radiography” OR “Panoramic Radiographies” OR “Orthopantomography” OR “Orthopantomographies” OR “Pantomography” OR “Pantomographies”)) Filter: English	33
Web of Science 20 June 2024	TS = ((“Artificial intelligence” OR “AI” OR “Computer Reasoning” OR “Machine Intelligence” OR “Deep Learning”) AND (“Dental Caries” OR “Dental Decay” OR “Dental Cavities” OR “Carious Lesion” OR “Carious Lesions” OR “Dental Cavity”) AND (“Panoramic radiograph” OR “Panoramic Radiography” OR “Panoramic Radiographies” OR “Orthopantomography” OR “Orthopantomographies” OR “Pantomography” OR “Pantomographies”)) Filter: English	8
Dentistry & Oral Sciences Source 20 June 2024	(“Artificial intelligence” OR “AI” OR “Computer Reasoning” OR “Machine Intelligence” OR “Deep Learning”) AND (“Dental Caries” OR “Dental Decay” OR “Dental Cavities” OR “Carious Lesion” OR “Carious Lesions” OR “Dental Cavity”) AND (“Panoramic radiograph” OR “Panoramic Radiography” OR “Panoramic Radiographies” OR “Orthopantomography” OR “Orthopantomographies” OR “Pantomography” OR “Pantomographies”) Filter: English	6

**Table 2:** Inclusion and exclusion criteria for screening articles.

Inclusion Criteria	Exclusion Criteria
The article must focus on panoramic AI and diagnosing dental caries. Peer-reviewed full-text original articles Articles written in English. Study involves human subjects. Studies published up to 2024.	Articles that are not related to panoramic AI and diagnosing dental caries. Literature reviews (narrative, scoping, systematic) Conference proceedings Editorials and opinions Articles that provide abstracts without the full text. Articles not written in English. Study involves nonhuman subjects.

**Table 3:** Summary of the advantages and disadvantages of each AI program.

Author Names	AI Program	Setting	Study Population	Advantages	Disadvantages
Asci, E. et al., 2024 [19]	Deep Learning Model (DLM)	Ataturk University, Turkey	Evaluated 6075 X-rays of children 4 to 14	The AI framework discussed in this study excels in identifying and classifying different dental conditions, including complex cases. AI offers strong integration with existing dental software systems, streamlining workflows in clinical settings.	Training the model requires a vast and diverse dataset, which may not always be available or easy to gather. There may be limitations in real-time diagnostics due to the processing time required for high-quality image analysis.
Başaran M., et al., 2021 [20]	CranioCatch	Eskişehi, Turkey,	Evaluated 1084 radiographs	High accuracy in specific tasks: The system excels in detecting dental conditions such as crowns, implants, and impacted teeth, with sensitivity values over 96%. Efficiency in diagnostic support: This AI tool can automate much of the diagnostic process, saving clinician time, especially during the initial analysis of panoramic radiographs. Potential for education and remote diagnostics: The system could assist in digital dental education and support remote diagnosis.	Inferior performance on certain conditions: AI struggles with detecting dental calculus and caries, showing low sensitivity (0.30 for caries and 0.09 for calculus), which could lead to missed diagnoses in these areas. Limited precision in some areas: The system shows lower precision for conditions like residual roots.
Dayı B., et al., 2023 [21]	DCD-Net (Dental Caries Detection Network)	Inonu University, Turkey	Evaluated 504 X-rays of individuals aged 14 to 80	Enhanced accuracy for lesion detection: This system uses deep learning for the precise segmentation of different types of caries on panoramic radiographs, improving diagnostic accuracy compared to traditional methods. Potential for early intervention: By detecting caries at various stages, it helps dentists to intervene earlier, which could lead to better patient outcomes.	Complexity of segmentation tasks: Although it is highly effective, it requires substantial computational resources and high-quality data for training, which might not be readily available in all clinical settings. Generalization issues: Like many AI models, its performance may decline when applied to different datasets that it was not trained on, limiting its general applicability.
Mărginean A.C., et al., 2024 [22]	CariSeg	Tufts U. (USA), Noor Center (Qom, Iran), Iuliu Haţieganu U. (Cluj-Napoca, Romania)	1116	The AI model presented in this article improves accuracy in diagnosing dental conditions through panoramic radiographs. The system offers rapid processing and can manage large datasets, making it scalable for clinical applications. It aids in reducing human error by providing consistent and reproducible diagnostic outcomes.	The system may have challenges with image variability due to differences in radiograph quality or patient positioning, affecting accuracy. Over-reliance on AI may reduce the dentist’s critical role in diagnosing complex cases that require a nuanced understanding of clinical context.
Y.C.C. Wang, et al., 2021 [12]	Diagnocat	Radboud University Medical Center (Radboudumc), Nijmegen Federal University of Minas Gerais (UFMG) National Taiwan University Hospital (NTUH)	6659	The learning model is highly effective in improving diagnostic confidence, providing reliable assistance to clinicians. AI’s ability to adapt to different image types and settings makes it versatile for a variety of dental diagnostics beyond caries detection.	The complexity of the model requires significant computational resources, which may limit its use in smaller or less-equipped clinics. Ethical concerns around AI decision-making in healthcare, particularly in terms of transparency and accountability.
Zadrożny, Ł. et al., 2022 [23]	Diagnocat	Medical University of Warsaw, Department of Dental & Maxillofacial Radiology	Ninety X-rays evaluated, 16 Male, 14 Female	The model has high specificity for apical periapical pathology, specifically apical periodontitis. Unacceptable reliability for caries detection. The Interclass correlation coefficient was 0.681, where > 0.75 was considered acceptable.	Was not able to diagnose periapical cysts, intermaxillary cysts, or broken endodontic instruments, though was able to identify these pathologies as abnormal. The model has low sensitivity for periapical pathology, specifically apical periodontitis.
Zhou X., et al., 2022 [24]	Tooth Type Enhanced Transformer (TTET)	Beijing Children’s Hospital, Beijing Stomatological Hospital, CAS, and Tsinghua University	6028	The model emphasizes interpretability, allowing dental professionals to better understand how the AI reached its conclusions. It shows high accuracy in detecting lesions and caries in radiographs, improving early detection rates.	The model might struggle in edge cases where the visual characteristics of a dental issue are ambiguous or differ from the training data. Implementation in real-world settings may face technical challenges due to integration issues with existing dental infrastructure.

**Table 4:** Specificity, accuracy, and F1 scores for each AI program.

Author Names	AI Program	Specificity	Accuracy	F1 Score	Number of Radiographs
Asci, E. et al., 2024 [19]	Deep Learning Model (DLM)	0.95	0.98	0.92	2785
Dayi B., et al., 2023 [21]	Dental Caries Detection Network (DCDNet)	0.48	0.56	0.61	504
Başaran M., et al., 2021 [20]	Craniocatch	0.30	0.51	0.38	1084
Mărginean A.C., et al., 2024 [22]	CariSeg	0.51	0.81	0.650	150
Y.C.C. Wang, et al., 2021 [12], Zadrożny, Ł. et al., 2022 [23]	Diagnocat	0.86, 0.98 Mean = 0.92 SD = 0.08	0.88, 0.85 Mean = 0.87 SD = 0.02	0.86, 0.59 Mean = 0.73 SD = 0.19	400, 30
Zhou X., et al., 2023 [24]	Tooth Type Enhanced Transformer (TTET)	0.83	0.880	0.87	210

## Data Availability

The original contributions presented in the study are included in the article. Further inquiries can be directed to the corresponding author.
